# Outcome of Streptomycin-Rifampicin treatment of Buruli Ulcer in two Ghanaian districts

**DOI:** 10.11604/pamj.supp.2016.25.1.6203

**Published:** 2016-10-01

**Authors:** Florence Nzilanye Iddrisah, Dorothy Yeboah-Manu, Pricillia Awoh Nortey, Kofi Mensah Nyarko, Jones Anim, Simon Nyovuura Antara, Ernest Kenu, Fred Wurapa, Edwin Andrew Afari

**Affiliations:** 1 Ghana Field Epidemiology and Laboratory Training programme, School of Public Health, University of Ghana; 2 Ghana Health Service; 3 Noguchi Memorial Institute for Medical Research, University of Ghana; 4 Department of Epidemiology and Disease Control, School of Public Health, University of Ghana

**Keywords:** Survival, Streptomycin/Rifampicin, Buruli Ulcer, endemic

## Abstract

**Introduction:**

Buruli ulcer (BU) is an infectious skin disease, caused by Mycobacterium ulcerans, endemic in more than 30 countries worldwide especially Africa. Brong-Ahafo Region implemented WHO recommended daily treatment with streptomycin and rifampicin for eight weeks (SR8). Yet limited assessment of therapy exists. This study seeks to determine the outcome of SR8 therapy on BU in two endemic districts in Brong-Ahafo.

**Methods:**

Longitudinal study was done with laboratory confirmed Buruli ulcer patients selected consecutively and put on SR8. Patient follow-up involved daily administration of SR8 and Bi-Weekly monitoring of treatment in the form of measurement of wound size and taking photographs.

**Results:**

The mean age of participants was 34.6 ± 16.6 years with minimum and maximum ages of 10 to 65 respectively. Those in the 10-19year age group 13 (26%) were most affected. Majority, 26 (52%) had no formal education and 27 (54.0%) were peasant farmers. Thirty-eight (76.0%) had previously used traditional treatment. Forty completed treatment and of these, 28 (70.0%) healed completely and 12 (30. 0%) improved by 80%-90%. Duration of lesion before seeking healthcare (P =0.04), use of traditional treatment P < 0.001, clinical form of lesion P = 0.04, lesion category (p = 0.01), significantly affected healing. Mean time to healing, was 7.7 weeks (95% CI, 7.3 - 7.9).

**Conclusion:**

Though SR8 is effective in curing BU, late reporting, use of herbs and access to health care impeded wound healing. This calls for provision of accessible health care and education to improve early reporting.

## Introduction

Buruli ulcer (BU), caused by Mycobacterium ulcerans (m. ulcerans), is an infectious neglected tropical disease that affects the skin[1]. The disease is common in some parts of the tropics, particularly in West Africa and has been reported from over 30 countries in Africa, Southeast Asia, Australia, and South America[2]. It is often found in swampy humid areas or near lakes and rivers, but the exact mode of transmission is unknown. People affected by BU are children aged 15 years or younger, often living in remote rural areas with little or no access to health services. The disease often manifests as a painless nodule, a firm plaque, or an oedematous lesion which soon ulcerates[3]. Massive destruction of skin tissues and sometimes the underlying bones, often leads to debilitating complications. Many patients report late with extensive ulcers that require surgical excision followed by skin grafting[3]. This was found to be costly and inaccessible to poor patients in rural areas due to limited treatment centers and lack of surgical capacities[4]. Thus the socio-economic burden of the disease on the affected poor families and communities with limited health facilities was enormous[5]. Based on several studies[4], the WHO in 2004 recommended directly observed treatment with rifampicin and streptomycin, administered daily for 8 weeks (SR8) as a first-line treatment for all forms of the active disease of BU [6–9]. Ghana was among member countries that implemented this protocol and currently over 30 treatment centers in 6 regions including Brong Ahafo provide this treatment[10] There is limited information on the outcome of the combined antibiotic treatment of streptomycin/rifampicin on Buruli ulcer in Brong Ahafo region since the inception of SR8 treatment protocol in 2006. This study determine the treatment outcome and factors that affect treatment in two BU endemic districts in Brong Ahafo.

## Methods

**Study Design:** a longitudinal study carried out in two health facilities with recruitment from February 2010 to April 2010. Active community search was done to identify the cases.

**Study population:** patients with a laboratory diagnosis of M. ulcerans disease were recruited from the Dormaa municipal and Asutifi districts in the Brong Ahafo Region of Ghana. Communities and health facilities were informed about the study, and ethical approval was obtained from the Ghana Health Service Ethical Review Board. Recruitment.

**Inclusion criteria:** patients were included if they were aged ten years or older; met the WHO clinical case definition for M. ulcerans disease [11]; must be a resident and ready to remain in the study area during the period of follow-up.

**Exclusion criteria:** BU patients who were pregnant; under treatment with antibiotics and or herbal treatment; had history of leprosy, tuberculosis, liver, kidney, or hearing problems. Those who met the inclusion criteria and gave written consent and assent for those less than 18 years were recruited. On recruitment, every lesion from which a specimen was collected was photographed, and the size and features of the lesion(s) were measured and recorded. Participants were grouped according to whether the ulcer was ulcerative or non-ulcerative. The lesions were further classed as category 1, 2, or 3 depending on lesion size.

**Laboratory confirmation of Clinical Diagnosis:** we conducted two laboratory tests to confirm clinical diagnosis of all the suspected cases who reported. Two swabs were taken from skin lesions of those with ulcers and FNA was done for those with non-ulcerative lesions. Samples were preserved and transported to the Noguchi Memorial Institute for Medical Research (NMIMR) for microscopy and Polymerase Chain Reaction (PCR). A positive case was defined as one that was PCR positive. PCR was repeated if a sample tested AFB+ but PCR negative. PCR was the Gold standard in this study.

### Definitions of Treatment Outcomes

**Successful treatment:** treatment was considered “successful” when the lesions were completely healed (scarred without residual inflammation) by completion of the 8-week antibiotic treatment without surgical excision.

**Loss to follow-up:** patients were considered as “lost to follow-up”(defaulters) when they abandoned or refused treatment and could not be traced.

**Failed treatment:** treatment was considered to have “failed” in the event of death related toi M. ulcerans disease and/or the persistence of non-scarring lesions despite appropriate medical treatment after SR8 treatment.

**Improved on treatment:** participants whose wound looked clean, the lesion has reduced 50% or more after SR8 treatment

**Treatment and treatment assessment:** All enrolled participants, regardless of the clinical form of the disease exhibited were put on daily treatment with a combination of rifampicin at 10mg/kg body weight by the oral route and streptomycin at 15 mg/kg body weight by intramuscular injection. Patients who were able to return each day to the clinic were treated on an ambulatory basis under the direct observation of the clinic nurse. Patients, who were unsuitable for ambulatory treatment, either because of the distance from their home to the clinic or because of the severity of the disease, were offered treatment through daily visits by the team. Every 2 weeks during treatment, the researcher with the help of the research assistants and medical officer evaluated each patient. During each evaluation we took fresh photographs of the lesions, measured the size and features to assess the evolution of the disease. Assessment of clinical improvement or deterioration was based on changes of the size of lesions, the degree of oedema, and the appearance of the ulcer base. Any increase in size, persistence of oedema, and/or absence of cleaning of the ulcer base was considered as an indication of little or no improvement. In addition, patients were questioned about symptoms of dizziness and abnormal hearing and examined for jaundice. On completion of four weeks of treatment, fresh specimens were taken again for laboratory examination to determine the presence of M. ulcerans. Upon completion of 8 weeks of antibiotic treatment, patients whose lesions were healed were included in a survival analysis. Surgical excision of the lesion was recommended for those patients whose lesions have not improved or have even worsened between weeks 4 and 8 of chemotherapy.

**Data Collection and analysis:** standard forms BU01 and BU02 were used to collect data. Data was analyzed using SPSS software Survival curves, and bivariate results were displayed using survival analysis and chi-square tests. Patient who had surgical excision together with those who were lost to follow-up and those who stopped as a result of adverse effects were censored from the survival analysis.

## Results

**Figure 1:**
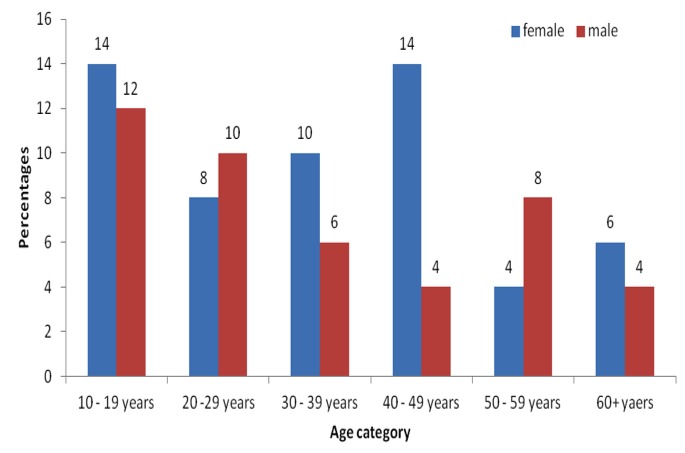
Age categories by sex distribution of 50 buruli ulcer patience put on rifampicin - streptomycin treatment in two endemic districts, Ghana, 2010

**Figure 2:**
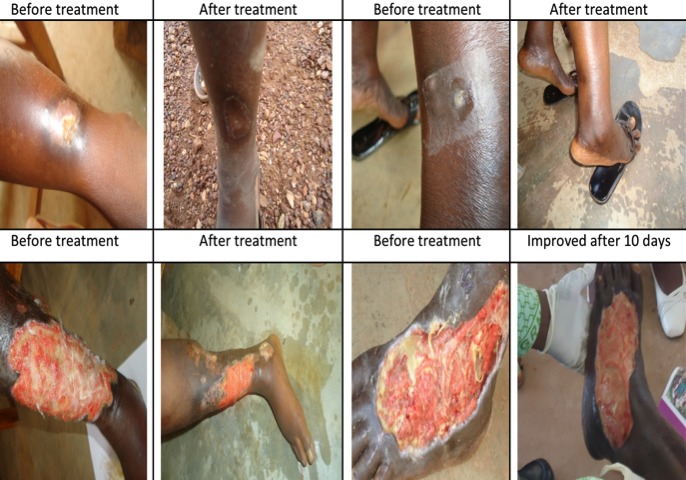
Buruli ulcer lesions before and after treatment with streptomycin/ rifampicin in two endemic districts, Ghana, 2010

**Demographic characteristics of participants.** All 50 participants who were selected for the study microbiologically tested positive for BU. Of these, the affected ages ranged from 10 years to 65 years with a mean age of 34.6 years and a Standard Deviation of 16.6. Most affected age groups were the 10-19 year age groups accounting for 13 (26%). Females accounted for 28 (56%) of all the study participants (Figure 1). Majority of respondents, 26 (52%) had no formal education and were mostly farmers 27 (54.0 %). Of those who did not have any form of employment, 9 (18.0%) were still in school. Classification of participants according to the Category (cat) of lesions yielded the following results; cat.1 were 15 (30%), whiles cat. 2 and cat. 3 lesions were 28 (56%), and 7 (14%) respectively. Of the total participants, 17 (34%) had a history of Buruli ulcer, 25 (50%) had trauma preceding the onset of lesion. Participants who had visible BCG scar were 32 (64%). Of all the cases, 38 (76%) used herbal treatment before seeking health care (Table 1). At the end of treatment, 40 (80%) completed treatment. Of the number that completed treatment, 28 (56.0%) got healed. Sequentially, wound healing was observed from week four by which 4 (15.4%) wounds had healed, 9 (30.8%) at week six and 15 (53.8 %) at treatment-week eight. The remaining 12 (15%) who completed treatment but did not heal, the ulcers improved dramatically and were proposed for surgery. The rate of remission for those ulcers that did not heal was 80% - 98% of the original size before beginning treatment. Buruli ulcer lesions before and after treatment with streptomycin/rifampicin in are shown in Figure 2. In logistics regression, risk factors such as age (p=0.06), sex (p=0.77), occupation (p=0.53), BCG immunization (p=0.38) and level of education (p=0.29) showed no significance in affecting healing. However, duration of lesion before seeking healthcare (P =0.04), use of traditional (herbal) treatment (P < 0.001) were found to significantly delay healing. The final regression model showed that those who used traditional medicine compared with those who did not were 3.0 times less likely to achieve healing. P<0.001, (95% CI, 0.18-0.34). Test of equality of survival distributions for the different levels of category of lesions showed that category one compared to category two and three has a relatively shorter meantime to healing of 7.2 (95% CI, 6.32-8.07). The Log Rank Test of equality of survival distributions for the different levels of use of traditional medicine by participants showed there was a significant difference in the mean time to healing between the levels of use of traditional medicine p < 0.001. Those who did not use traditional medicine compared to those who used have a shorter time to healing with mean healing of 6.9 (95% CI, 6.1-7.8) compared with 8.0 (95% CI, 8.00-8.00) for those who used traditional medicine.

**Table 1:** category of lesion by Age, sex, site, and type of lesion for 50 participants with *M. ulcerans* disease

Category	No. of participants	Complete treatment		Healed		Lost to follow-Up		Use herbs		BCG scar	
	(%)	Yes	No	Yes	No	Yes	No	Yes	No	Yes	No
Category 1	15 (30.0%)	12	3	12	3	3	12	10	5	8	7
Category 2	28 (56.0%)	23	5	16	12	5	23	23	5	19	9
Category 3	7 (14.0%)	5	2	0	7	2	5	5	2	5	2
Total	50 (100%)	40	10	28	22	10	40	38	12	32	18

## Discussion

The main objective of this study was to assess the effect of streptomycin/rifampicin (SR8) treatment on the healing of BU and if there are hindrances to the protocol. Most cases were within the group 10 - 19 years of age. It is consistent with study results that M. ulcerans is seen mainly in children and young adults in other BU-endemic regions but may affect any age group. A similar pattern has been reported in Ghana[12]. In our study, the females were more affected than the males. However, there were no significant differences among the age and sex distribution of cases (p = 0.62). This compares with a study that found no significant difference in the sex of respondents[13]. There was no significant deference in the healing of an ulcer with age, sex, and occupation. In the present study, ulcerative forms accounted for most of the cases, an evidence of late reporting. Our finding is in line with several studies that reported more ulcerative forms of the disease than the pre-ulcerative forms reported. Likewise in Benin, 72% of cases were in the ulcerative stage in the surveillance data analysis done and another study on risk factors reported 71% of ulcers in their risk factor study [14]. The present study showed that most of the cases sought herbal and traditional treatment and reported to health facility when the disease had progressed. The present study found that the use of herbal treatment (p <0.001), were significant in delaying healing. It was found out that people who used herbal medicine were 5.8 times less likely to achieve healing compared to those who did not (95% CI, 0.00-0.23). This could be as a result of the cultural believes about the cause of Buruli ulcer and the superstition attached to the disease. Hence affected people tend to look for spiritual help as a primary step. The observation of sociocultural belief influencing the timely reporting of BU patients to health facilities[15, 16] was also observed in our study. The findings also emphasize the need for education on the importance of early reporting to the formal sector to seek help. Our study found that streptomycin and rifampicin therapy is effective in the treatment of BU as evidenced by the treatment success of those who remained in the programme to the end of the eight weeks duration. Our study showed that about half of the participants who completed treatment got healed and there was marked improvement in those who did not achieve complete healing after eight weeks of treatment. Overall, these findings confirm the potent bactericidal activity of the 2-month course of streptomycin-rifampicin demonstrated experimentally in the mouse footpad model[17] and in the clinical trial conducted in Ghana and in Benin[4]. The lack of alternatives to the streptomycin-rifampicin combination was undoubtedly a hindrance to the successful completion of the 8 weeks treatment regimen for those who experienced side effects of the drugs given. Even though they were transient and resolved after some time, the affected participants were reluctant to continue treatment. Time was a limiting factor in the study. We could not extend the follow-up period beyond eight weeks for the outcome of those who improved but could not achieve healing in eight weeks. The sample size of fifty participants was relatively small however, it confirms the efficacy of the treatment and identified some factors that affect treatment success. Moreover treatment outcome does not rely on numbers and most studies on BU have fewer numbers but gave accurate results.

## Conclusion

This study validated that SR8 is effective in healing early lesions and improve ulcers dramatically. However, the use of traditional medicine was an important factor affecting healing. Healing for early lesions and for those who did not use herbs was reduced to about six weeks compared to those with late lesions and or used herbs respectively. SR8 proved effective for those who remained in the study irrespective of the state and lesion category. Buruli ulcer of all categories can improve dramatically with streptomycin and rifampicin if clients report early, adhere to treatment and do not use herbs before treatment. This study results stresses the need to intensify education in the control of BU in Ghana. In addition there is the need to test interventions that could improve early reporting such as resources for active case search and enablers package. A study into factors that affect efficacy and adherence to treatment of Buruli ulcer with streptomycin/rifampicin is recommended.
